# Hydration Effects Driving Network Remodeling in Hydrogels during Cyclic Loading

**DOI:** 10.1021/acsmacrolett.4c00653

**Published:** 2025-01-27

**Authors:** Baptiste Le Roi, Joshua M. Grolman

**Affiliations:** Materials Science and Engineering Department, Technion-Israel Institute of Technology, Haifa 3200003, Israel

## Abstract

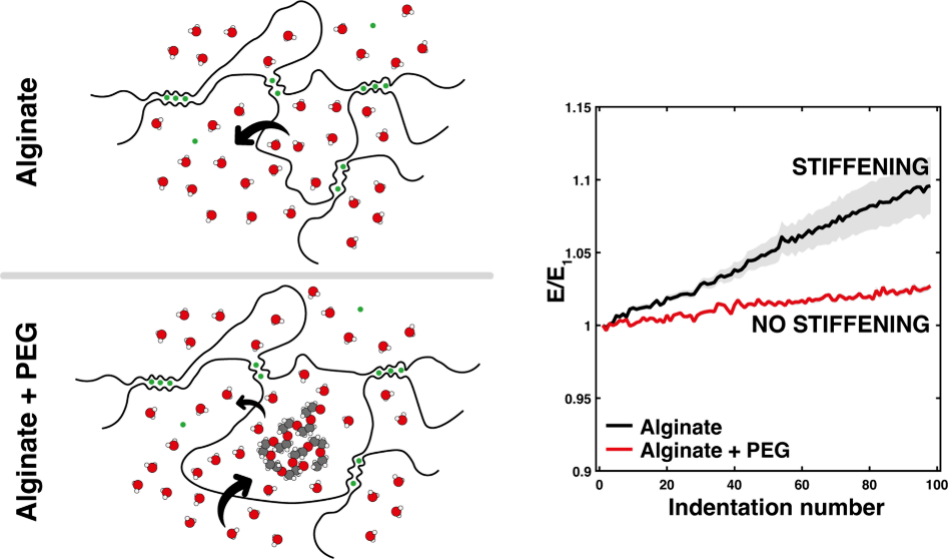

In complex networks and fluids such as the extracellular matrix, the mechanical properties are substantially affected by the movement of polymers both part of and entrapped in the network. As many cells are sensitive to the mechanical remodeling of their surroundings, it is important to appreciate how entrapped polymers may inhibit or facilitate remodeling in the network. Here, we explore a molecular-level understanding of network remodeling in a complex hydrogel environment through successive compressive loading and the role that noninteracting polymers may play in a dynamic network. We find that this is a highly localized and time-dependent effect, with one of the major driving factors of hydrogel matrix remodeling the interaction and movement of water within the network in calcium-cross-linked alginate. Our results suggest a more general mechanistic understanding of hydrogel remodeling, with implications for tissue transformations in disease, biomaterials, and food science formulation.

## Introduction

Hydrogels are found in the food we eat and the makeup we wear, and even much of our bodies consists of hydrogels. These networks often contain incredibly complex mixtures of polymers, some of which interact chemically,^1^ sterically,^2^ or through solvation effects.^3^ Polymers that do not have chemical or steric interactions within networks play enormous roles in food processing,^4^ remodeling of tissue in either health or disease,^5^ and biomedical applications,^6^ where they can act as viscosifiers,^7^ emulsifiers,^8^ or plasticizers.^9^

Their roles in regulating the mechanical properties of the larger network have been explained mechanistically with free volume theory, where they modulate the contact, or free volume, between close polymer chains and therefore dictate chain mobility.^10^ In the case of plasticizers, this often accompanies a decrease in the glass transition temperature (*T*_g_) of the host polymer, enhanced elongation and flexibility of the bulk material, and a decrease in the Young’s modulus.^11^ Yet, what often is overlooked is the role hydration plays.

Water has been shown to be a significant plasticizing element in a variety of hydrophilic polymers,^12^ though this is often in the context of the relative humidity of hydrophilic thin films for packaging.^13^ For hydrogels, networks that are hydrated and immersed in water, it is assumed that the interactions with water are fully saturated, and therefore any further addition of a hydrophilic polymer will not have a significant impact.^14^ Polymers like poly(ethylene glycol) (PEG) fit this role classically, as it has been used on numerous occasions as a noninteracting humectant and as a plasticizer.^15^ However, this was usually measured in elongation during uniaxial tensile or compression testing in water-conserved methods.^16^ The question then remains, what is the effect of encapsulating a hydrophilic polymer like PEG inside of a similarly hydrophilic network like alginate; a situation akin to encapsulated proteins in the extracellular matrix (ECM)? This hydrophilic modification effect has been observed in covalently modified protein based gels^17^ and poly(phosphoester) gels^18^ previously; yet for encapsulated proteins, the effect is largely unknown.

For cells, the ECM is a comparable network filled with such encapsulated proteins and has been modeled with calcium-cross-linked, covalently bound PEG-alginate hydrogels^19^ that demonstrated control over stress relaxation, and as a result, regulation of cellular phenotype and genotype.^20,21^ Though PEG was often claimed as a plasticizer,^15^ the mechanism has not been explored. Previous alginate-PEG hydrogels did not consider the addition of PEG as a structural component, rather as a modifier of network plasticity or stress relaxation behavior.^22^ This often took the form of covalent coupling of the PEG to the alginate, resulting in significant decreases in the relaxation half-time during stress relaxation tests and dramatically affecting the encapsulated cell phenotype and genotype.^23^ In this work, we prepare an alginate hydrogel as a model environment for successive compressive localized strain in order to investigate what may be driving the polymer network transformation. With matrix remodeling of the polymeric surroundings of cells, changing their volume,^24^ playing pivotal roles in various diseases, like myelofibrosis,^25^ it is important to understand how the remodeling is occurring to develop diagnostic tools and therapies.^26^

## Results

### PEG Stiffens Alginate Hydrogel Networks

Though previous work has controlled for Young’s modulus in calcium-cross-linked alginate PEG hydrogels,^23^ we wanted to investigate the effect of PEG polymer as a fractional mass of the total polymer load. PEG addition, both encapsulated and covalently incorporated as pendant groups, has not shown significant increases in the Young’s modulus previously at up to a 10% mass of alginate, which suggests its limited interaction with the calcium ion cross-linking mechanism.^23^ PEG was also chosen with a size of 10 kDa, or approximately 7 nm as measured by Zetasizer (Figure S1), in order to be well below the average pore size of alginate hydrogels, which can range from 16 nm^27^ to hundreds of nanometers.^28^ To investigate domains where encapsulated PEG might impact the Young’s modulus of the resulting hydrogel, samples were prepared with a significant excess of calcium ions to ameliorate any effects that vacant binding sites may have on the cross-linking and remodeling in the hydrogel in response to deformations. As the mannuronate to guluronate (M/G) ratio of the Kimica 1G alginate was determined to be 46:54 via FTIR (Figure S2),^29^ the minimum saturation concentration was calculated as 1.2% (w/v) CaSO_4_ (Figure S3). As we prepared our gels in accordance with previous studies,^30^ the concentration of CaSO_4_ was 3% mass, which was well above the saturation levels required.

By increasing the amount of encapsulated PEG while maintaining the mass of alginate and calcium constant, we can ascertain the effect of encapsulation on Young’s modulus.

**Figure 1:**
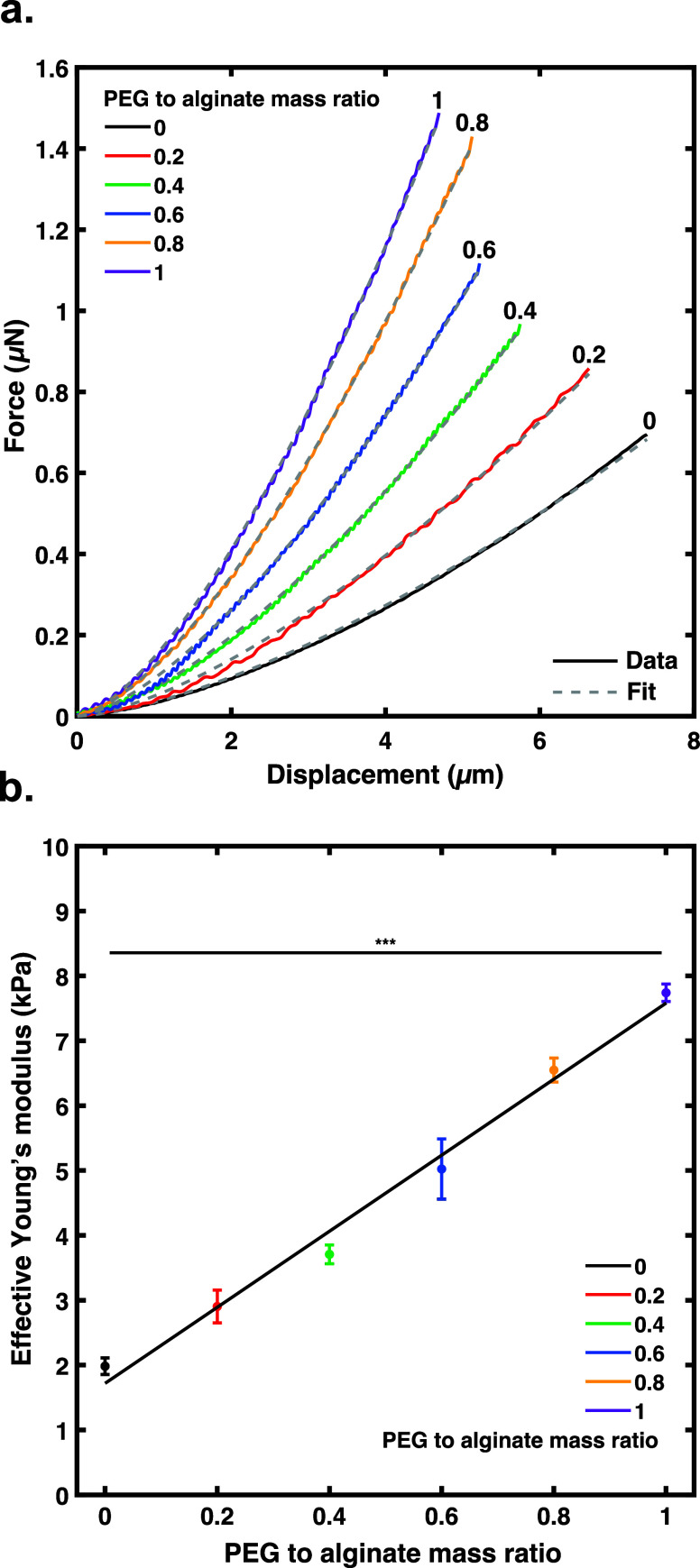
(a) Representative force–displacement compressive indentation curves for alginate-PEG hydrogels with a 100 μm polypropylene spherical probe and increasing PEG content as a ratio of the alginate polymer mass (0, 0.20, 0.40, 0.60, 0.80, and 1.0) and their associated fitting with a Ding model (dashed lines). (b) Effective Young’s modulus average values as calculated via fitting the Ding model to the entire force–displacement signal using the Levenberg–Marquardt nonlinear least-squares algorithm, *n* = 15.

The mass ratio was increased from 0 (no PEG added) to 1 (equal mass of PEG added). This was demonstrated by a progressive increase in the slopes of the force–displacement curves, as measured by steady state nanoindentation in the elastic regime (Figure 1a). The tests were performed in an HBSS calcium and magnesium-free bath with a 145 mM NaCl salt concentration to limit the effects of swelling in the immersion bath during testing. Though the vertical displacement was relatively small, local strains may be quite high, particularly at the central interface between hydrogel and the probe. As a result, the experimental load–displacement curves were fitted with the Ding model (eq S1) in order to achieve better conformation under larger strains due to hyperelastic considerations,^31^ especially compared to the Hertz model (eq S2) fit.^32^ Like Hertz, the Ding model does not take into account viscoelasticity, but at a loading rate of 5 μm s^–1^, the system should already be at a steady state. Using this model, the effective Young’s moduli were calculated for each mass ratio of encapsulated PEG.

The addition of noninteracting PEG led to surprisingly significant increases in the effective Young’s modulus of calcium-cross-linked hydrogels. This was despite PEG not participating in cross-linking, carrying a nearly neutral ζ-potential of −10 mV measured by Zetasizer,^33^ compared to alginate at approximately −21 mV (Figure 1b).^34^ It was also interesting to note that the trend was remarkably linear (*R*^2^ = 0.99) with effective Young’s moduli ranging from 2.0 kPa for neat calcium alginate gels to 7.6 kPa for gels with a PEG to alginate mass ratio of 1. These trends are usually correlated with increases in molecular weight, either through the polymer backbone or cross-link bond formation,^35^ and in this case, there should be neither of these options present.

### PEG Is Likely Not Chemically Interacting with the Hydrogel

**Figure 2:**
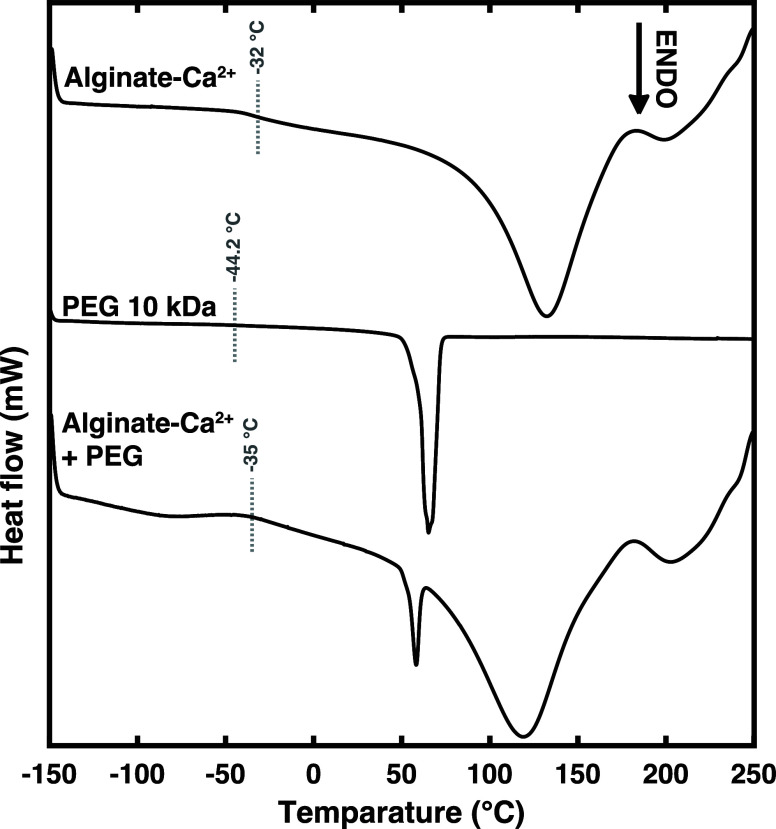
DSC thermogram curves of pure calcium-alginate gel and calcium-alginate + PEG (0.2 peg to alginate mass ratio) cross-linked hydrogels. The samples were lyophilized, ground, and then measured under nitrogen, frozen up to −150 °C, and heated until 250 °C at a heating rate of 10 °C min^–1^.

If the PEG was not interacting chemically, nor affecting chain mobility, then it may be observable through changes in thermal energy exchange.^36^ By performing scanning calorimetry measurements, the endotherms were explored of alginate, PEG, and cross-linked alginate with PEG. The samples cooled to −150 °C at a cooling rate of 10 °C min^–1^ and heated up to 250 °C at a heating rate of 10 °C min^–1^. There were distinct glass transition temperatures that corresponded with PEG at −44 °C (Figure 2)^37^ and 55.6 °C for neat alginate (Figure S4a).^38^ Upon cross-linking with calcium, the *T*_g_ then shifted to −35 °C (Figure 2), which was consistent with the literature.^39^ Upon adding the PEG prior to cross-linking, the data show that the corresponding alginate *T*_g_ of the combination of alginate, PEG, and calcium slightly shifts to −32 °C (Figure 2). Considering the margin of error, there were seemingly no significant shifts in the *T*_g_ of either component. This suggests that the addition of encapsulated PEG does not dramatically affect chain mobility or free volume due to chemical interactions like hydrogen bonding or electrostatic repulsion or attraction and is likely not acting as a plasticizer in these composite hydrogels in a traditional sense.^40^ With minimal changes in alginate mobility, the question then remains how PEG seems to increase the network modulus without participating in the cross-linking mechanism.

### PEG Drives Swelling of the Pores

The increases in effective Young’s modulus that correlated with the amount of PEG to alginate mass ratio may perhaps be explained by a swelling phenomenon. As PEG is notably more hydrophilic with a solubility constant of 300 mg/mL compared to 20–30 mg/mL at 23 °C for alginate,^41,42^ an increase in mass fraction would likely lead to increases in the swelling mass ratio. Prepared calcium-alginate-PEG hydrogels were cut into 9-mm-diameter circles 6-mm-thick and then placed in distilled water. The mass and the volume of the samples were measured before submersion in DI water and after 24 h. Surprisingly, all samples exhibited slight deswelling, although the effect was abrogated by increasing the encapsulated PEG in the network (Figure S5). Deswelling is a common phenomenon, especially at higher calcium ion concentrations, due to not only autocooperative binding between lateral chains but also the diffusion of surplus calcium sulfate and calcium ions into the immersion media.^43^ As the calcium was over the saturation levels in this case, this would explain the mass loss of all samples, despite PEG concentrations.

**Figure 3:**
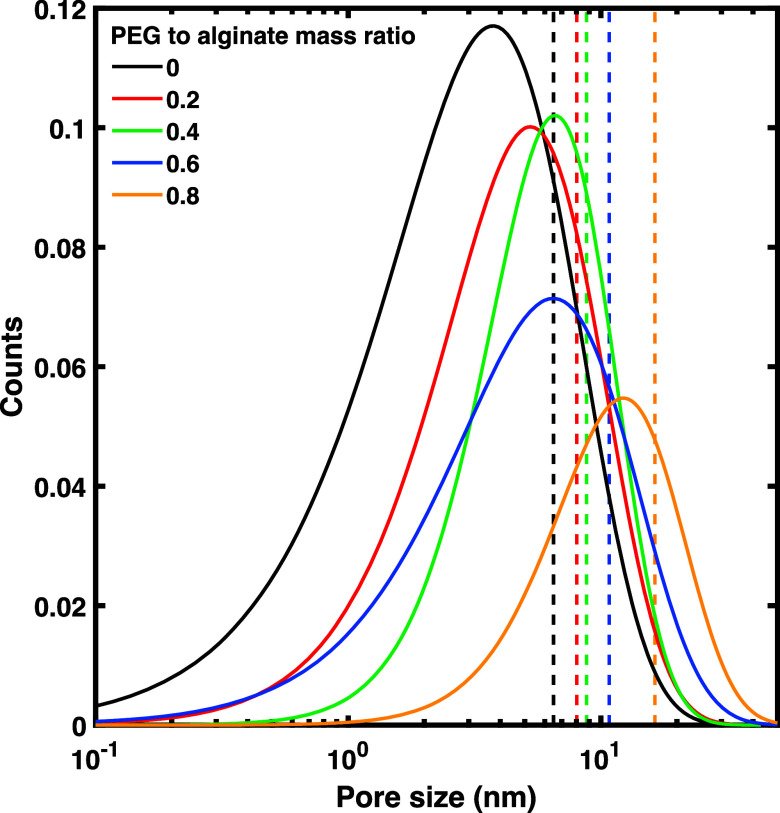
Pore size measurement with SAXS for alginate-PEG gel with PEG concentration varying from a 0 to a 0.8 PEG to alginate mass ratio after 24 h of immersion on water. Dashed lines correspond to the average pore diameter.

This agrees with the volumetric data showing that the hydrogels increased in volume simultaneously; so as surplus calcium and other ions were diffusing out, water was still diffusing in (Figure S5). However, the PEG loading appears to be driving this behavior, as water content at the time of hydrogel cross-linking has been shown to dominate the swelling behavior, up to a 0.6 PEG to alginate mass ratio. This also corresponded to an increase in the pore volume size, as calculated from small-angle X-ray scattering (SAXS) and scanning electron microscopy (SEM) (Figure S6). As demonstrated in Figure 3, the pores of the hydrogel network increased in cross-sectional size from 6.5 nm without any PEG, to 16.4 nm with a 0.8 PEG to alginate mass ratio.^44^ This seems linked to a decrease in the porosity with simultaneous increases in pore sizes, suggesting that the volume and overall structure-conserving aspects of calcium-cross-linked hydrogels remained unchanged. Therefore, as the pore size and hydrogel volume increased upon addition of PEG, this may be due to the retention of water, drawn into the pore and loosely associated with the PEG. If the movement of water was indeed causing swelling of the pores and, by extension, the elongation of polymer chains in between cross-linking regimes, then the effect of repeated compressions would lead to differences in viscoelastic behavior.

### Movement of Water Behind Hydrogel Remodeling

**Figure 4:**
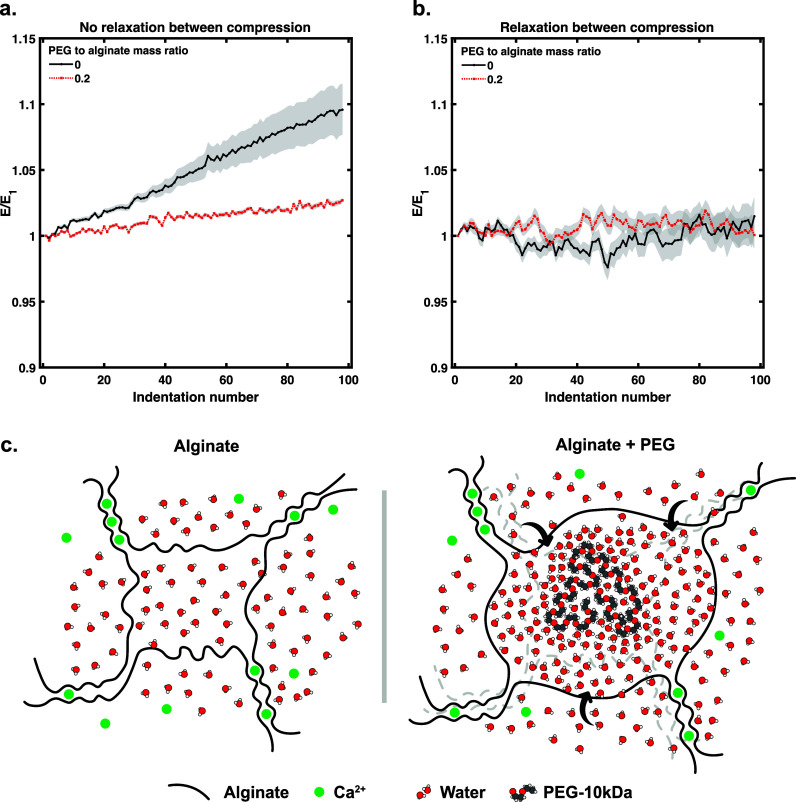
(a) Stiffness ratio (E/E1) evolution over 100 immediate indentation cycles at the same location for pure alginate and an alginate + 0.2 PEG to alginate mass ratio. (b) Stiffness ratio (E/E1) evolution over 100 delayed (three relaxation periods) indentation cycles at the same location for pure alginate and alginate with a 0.2 PEG to alginate mass ratio. (c) Molecular schematic of the hydration effect on polymer remodeling.

Viscoelastic properties are often thought of as dominated by the underlying polymeric framework of the hydrogel,^45^ and as such, the displacement of water within that network is often overlooked. By performing successive and relatively small compressive deformations with a nanoindentation probe, we can investigate how water may be displaced. It has previously been demonstrated that altering the viscosity of the aqueous phase of hydrogels can impact viscoelasticity,^46^ but in this case, the viscosity of water with or without such small amounts of PEG were not significantly different. A series of 100 steady state indent cycles were executed on a singular point on the hydrogel in displacement control mode. The speed of indentation was selected at 5 μm s^–1^, to be faster than the network relaxation time to probe in the predominately elastic regime, and in a regime where the effect on modulus to be minimal (Figure S7). The effective Young’s modulus was then calculated using the Ding model and normalized to the first modulus obtained. Figure 4a demonstrates the apparent strain-stiffening and viscoelastic properties of the alginate hydrogel at a ratio of encapsulated PEG of 0.2 that of alginate, where the effect is abrogated. This effect was observed for 0.4 and 0.6 ratios of encapsulated PEG as well (Figure S8). Interestingly, we allow a relaxation period three times the measured relaxation time (Figure S9), for a total of 15 s between each of the compressive loads; the differences in response between the alginate hydrogel with or without PEG disappear (Figure 4b). One explanation for this is that the PEG is slower to diffuse out of the mesh in response to small deformations,^47^ and so when some water is pushed out of the pore, it can be attracted right back in, therefore appearing more elastic in its response (Figure 4c). However, for the neat calcium-alginate hydrogel, there are less hydrophilic interactions drawing them back into the pore space.^48^ Therefore, when sufficient time is given for the hydrogel to relax in between successive compressive perturbations, it does not strain-stiffen in the same way and appears to lose its viscoelastic effects. This seems to be independent of the initial effective Young’s modulus, which is in contrast for static load versus cyclic loading associated with remodeling of the network.

## Conclusion

In summary, this work demonstrates that, for complex hydrogels containing nonchemically interacting polymers, their affinity for water may be contributing to both the intrinsic mechanical properties as well as the ability to remodel in a time-dependent manner. Ranging from increased rigidity due to swelling and enlarged pore size to even the viscoelastic response under successive loading, this should be a factor for consideration in the design of hydrogels for biomimicry of the ECM and other complex hydrogels where network remodeling and intrinsic mechanical properties can have profound effects.

## Supporting Information Available

Chemicals, gel preparation for nanoindentation, compression experiments and effective Young modulus calculation, nanoparticle size determination, alginate’s M/G ratio calculation via FTIR, pore size determination via SAXS, DSC signals, swelling ratio experiments, and gel relaxation tests (PDF)
