# Supplementary material for: Hydration Effects Driving Network Remodeling in Hydrogels during Cyclic Loading
Source: ACS Macro Lett. 2025 Jan 27;14(2):176–81. doi: 10.1021/acsmacrolett.4c00653 (PMC11841051; doi:10.1021/acsmacrolett.4c00653)
Supplement: Supplementary file 1 — mz4c00653_si_001.pdf [file mz4c00653_si_001.pdf]

Supporting Information for

Hydration effects driving network remodeling in hydrogels during cyclic loading

Baptiste Le Roi, Joshua M. Grolman*

Materials Science and Engineering Department, Technion-Israel Institute of Technology, Haifa, IL

* Corresponding Author: JMG: grolman@technion.ac.il

Chemicals

Sodium Alginate (#1-IG) was purchased from Kimica (Tokyo, Japan). Carboxymethyl-PEG-OH MW = 10 000 Da (CM-PEG-OH, #111820CE-01) was purchased from Laysan Bio (Arab, AL, USA). Calcium Sulfate (#237132) was purchased from Sigma Aldrich (St Louis, USA). HBSS without calcium, magnesium, and phenol red (#02-018-1A) was purchased from Biological Industries (Beit Ha Emek, Israel). The water used throughout all the experiments was purified from a Wasserlab water purification system (Automatic Plus Type I, Barcelona, Spain).

Gel preparation

To test the strain stiffening on gels two types of alginate gels were prepared: Alginate and Alginate-PEG gels.

Suspension preparation

HBSS-Alginate 1.6 % (w/w) was prepared by scaling 640 mg of unfrozen alginate powder, adding it into 40 g of HBSS and shaking it overnight at room temperature on an orbital shaker (Multi Reax, Heidolph, Germany) at 1100 rotations per minutes. HBSS-Alginate 1.6 %-PEG [0. - 1.6] % solutions were prepared by mixing 640 mg of alginate powder with between 128 mg (0.2 PEG to Alginate mass ratio) to 640 mg (1 PEG to Alginate mass ratio) of unfrozen CM-PEG-CH powder.

Then the powder mix was added to 40 ml of HBSS and shaken overnight at room temperature on an orbital shaker.

HBSS-$CaSO_4$ 3 % (w/w) slurry was prepared by mixing 1.2 g of $CaSO_4$ powder into 40 ml of HBSS at 600 rpm.[1] It is important to note that $CaSO_4$ was dried at least two hours at 150 °C prior solution preparation. HBSS-$CaSO_4$ 3 % (w/w) was prepared freshly for every gel synthesis.

Alginate and Alginate-PEG gels molding

Alginate and Alginate-PEG gels were prepared by rapidly pipetting four portions of a HBSS-Alginate 1.6 % suspension with one portion of HBSS- $CaSO_4$ 3 % slurry. This pipetting is done by connecting two syringes with a Luer-lock connector and pressing alternatively on one side and then the other to homogeneously mix the two suspensions. After seven cycles, the syringes are disconnected, and the mixture is placed between two glass plates 1 millimeter spaced from each other. Then, the gel is left at room temperature for half an hour to complete cross-link of the gel. Finally, the gel is unmolded from the glass plates and prepared for nanoindentation experiments. Alginate-PEG gels were prepared the same way as Alginate gels.

Nano indentation samples preparation

Nanoindentation experiments were done using an Optics 11 nano-indenter (Chiaro, Optics 11 For Life) and run in a liquid environment. As alginate gels float, it is mandatory to immobilize them firmly. To do so, agar 4 % solution was prepared by dissolving 4 g of agar (Bacto Agar # 214010, Becton Dickinson, US) into 100 ml and heating it at 95 °C with mild stirring until no clumps were visible. Meanwhile, 9 mm diameter alginate discs were punched out of alginate films with a circular puncher. Approximately 2 ml of agar solution was poured into a 60 mm diameter Petri dish, and alginate discs were gently placed before the agar solidified. Finally, a joint of agar was placed around the alginate disk by extruding liquid agar with a one-time-use pipette. This seal prevents the alginate disc from floating and limits the presence of water between the sample and the substrate, which might impede proper measurement.

Graphing and statistics

All plots were plotted using Matlab (R2024a, MathWorks, USA), error bars corresponding to the SEM of the data; However, numbers in text are given in the format mean (Standard Deviation). Statistical tests (one way ANOVA) were calculated using OriginPro 2023b (Ver 10.0.0.154 academic, OriginLab Corporation, US).

PEG size determination

To verify that the PEG particles would fit into the alginate cell lattice, we determined the hydrodynamic radius and the zeta potential of a 0.1 % (w/v) peg in water solution using a Zetasizer Nano ZSP, Malvern PANalytical dynamic light scattering device (DLS). The device uses a 633 nm He-Ne laser, at a scattering angle of 173 °, at room temperature. We took the values given by the Zetasizer Software version 7.04 that uses the Stokes-Einstein equation (Figure S1). We found three nanoparticle dimensions, 60.8 (±5.7) % of them has a hydrodynamic radius of 7 (±0.45) nm and others thirty-four time bigger 238 (±50) nm (36.3 (±3.8) %). PEG nanoparticles are considered neutral as the zeta potential value at -10.2 (±0.5) mV.[2]

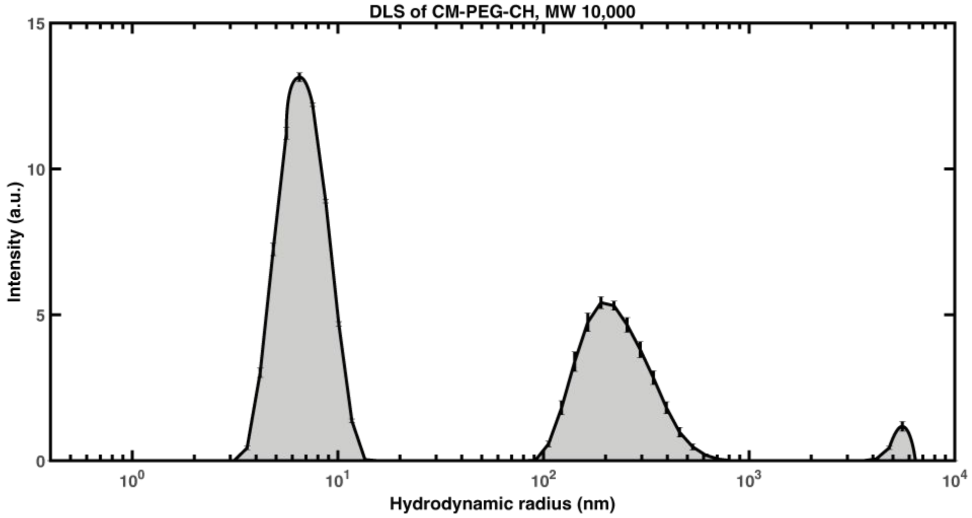

Figure S1: DLS of PEG, 60 % of the particles are about 7 mn diameter.

M/G ratio calculation and bonding equivalent sites number in the gel

M/G ratio of the alginate used was estimated following the method of Sartori et al.[3] First thin films of alginate were prepared, then measured by FTIR. The M/G ratio was determined by the ratio of absorbance at 1290 cm^{-1} (mannuronate contribution) and 1320 cm^{-1} (glucuronate contribution).

Film preparation

First, a 1 % mass alginate suspension was prepared by suspending 250 mg of alginate into 25 ml of deionized distilled water and shaken over night at room temperature. Once fully suspended and clear, the suspension was setting 20 min under vacuum to remove bubbles obtained during shaking, then few milliliters of it were poured into 30 mm diameter petri dishes and dried in an oven set at 50 °C during 2 hours. In the meanwhile, a 0.8 % (w/v) calcium chloride solution was prepared by dissolving 800 mg of calcium chloride (# 0340, Biosolve, France) into 100 ml deionized distilled water. Then to cross-link alginate films, 5 ml of the calcium chloride solution was poured on top of the dried alginate film and set at least 30 min in room temperature for complete curing. Finally, the calcium chloride solution was removed and cross-linked alginate films were dried a second time at least 2 hours in an oven set at 50 °C. The films were 200 µm thick.

Fourier-Transform Infrared spectroscopy (FTIR) experiments

Infrared spectra were acquired with a Nicolet iS50 FTIR spectrometer (# 912A0760, Thermo Scientific, US) from 550 to 4 000 cm^{-1} and averaged over 128 scans at a resolution of 4 cm^{-1}. Prior to measuring the sample, a blank measurement was done on air. The spectra were pre-processed with OMNIC software version 9 by first normalizing the absorbance and then manually setting the baseline.

FTIR Spectra analysis

Spectra were analyzed on Matlab, the M/G ratio is defined as the ratio of absorbance at 1320 and 1290 cm^{-1} then the mass proportion φ_M and φ_G of M and G monomers were calculated as follows:

$$\begin{cases} \varphi_M/\varphi_G = \dfrac{A_{1290}}{A_{1320}} \\ \varphi_M + \varphi_G = 100 \end{cases}$$

M/G ratio has been calculated on five samples.

Results

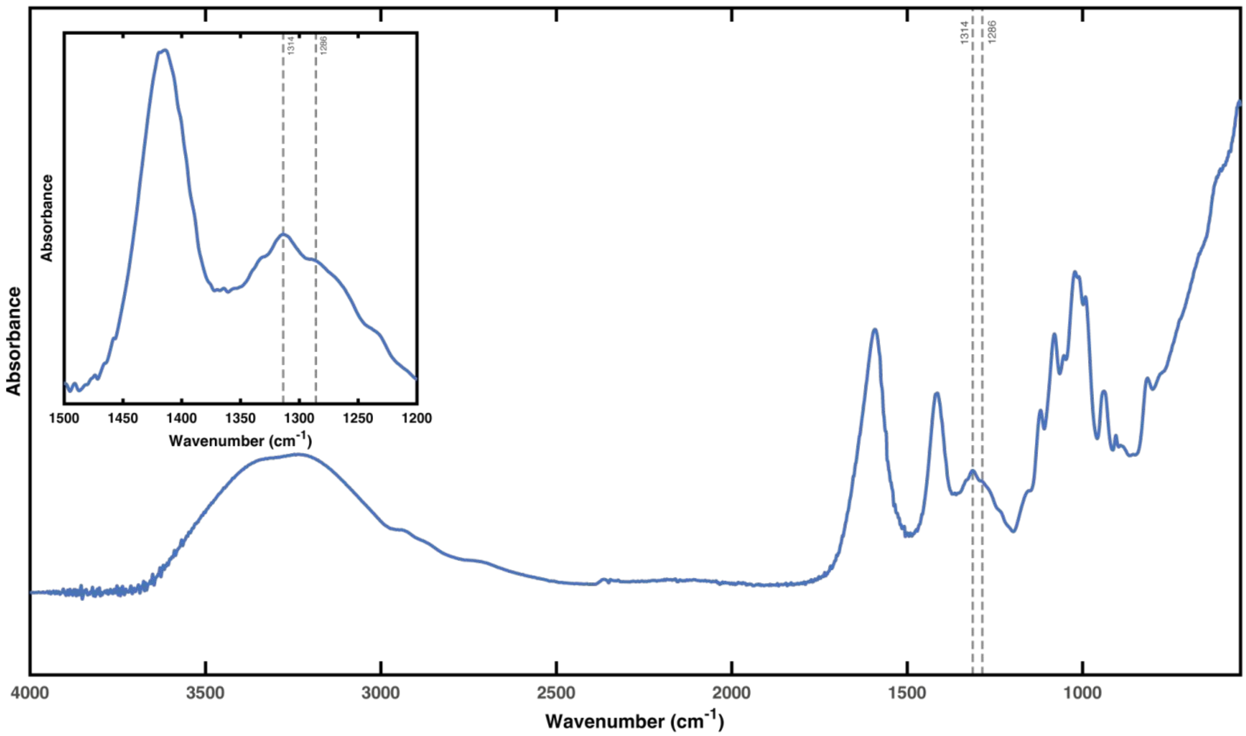

Figure S2: FTIR spectrum of 1-IG alginate 1.6% solution in HBSS cross-linked with a $CaCl_2$ 1 % (w/v) solution.

The M/G ratio for Sodium Alginate (#1-IG, Kimika, Japan) is about 46:54 (**Figure S2**), which corresponds to a medium M / medium G alginate gel. In addition, the molecular weight has been determined previously by viscosimetric analysis (Mark-Houwink law): Number average molar

mass, M_n = 84.2 kDa, and Mass average molar mass, M_w = 125 kDa and thus a dispersity of I = 1.49.[1]

Number of equivalent bonding sites in the alginate

Stiffness of the alginate gel is mainly due to the number of the calcium cross-links, in order to better understand the effect of varying the calcium concentration on mechanical properties, we want to determine the ratio of calcium saturation.

It is commonly accepted that one calcium ion binds with four carboxylic acids of glucuronate, two from one chain and two from another alginate chain.[4] Knowing the fraction of G monomer in the alginate polymer it follows that determining the potential number of bonding sites N as

$$N = \frac{1}{4} n_G$$

where n_G is the number of G monomers (mol). Luckily, glucuronate and mannuronate have the same molar mass $M_G = M_M = 194.139\ g.mol^{-1}$. So the number of G monomer n_G per chain corresponds to:

$$n_G = \phi_G \frac{M_{w_{Alg}}}{M_G}$$

where ϕ_G is the fraction of G monomers in the alginate, $M_{w_{Alg}}$ is the molar mass of the alginate ($g.mol^{-1}$), and M_G the molar mass of glucuronate monomer. Knowing n_{Alg} the number of chains in the gel (mol), the number of bonding sites becomes

$$N = n_{Alg} \cdot \frac{1}{4} \cdot \phi_G \frac{M_{w_{Alg}}}{M_G}$$

Finally, we can easily express the number of alginate chains in function of m_{Alg} the mass of alginate (g), $C_{p_{Alg}}$ the mass concentration of alginate (g.l^{-1}), and V_{Alg} the volume of alginate solution used to prepare the gel (l). The number of bonding sites becomes

$$N = \frac{1}{4} \cdot \frac{m_{Alg}}{M_{w_{Alg}}} \cdot \varphi_G \frac{M_{w_{Alg}}}{M_G} = \frac{1}{4} \cdot \frac{C_{p_{Alg}} \cdot V_{Alg}}{M_{w_{Alg}}} \cdot \varphi_G \frac{M_{w_{Alg}}}{M_G} = \frac{1}{4} \varphi_G \cdot \frac{C_{p_{Alg}} \cdot V_{Alg}}{M_G}$$

where n_{Alg} is the number of alginate molecules (mol), and m_{Alg} is the mass of alginate (g). For a gel made of 2 ml of a 1.6 % alginate solution, the number of potential bonding sites is about

$$N = \frac{1}{4} * 0.54 \frac{16 \cdot 0.002}{194.139} = 22 \text{ µmol}$$

Saturation of calcium sulfate

We want now to determine the $CaSO_4$ solution concentration that will saturate 2 ml of alginate 1.6 % (w/v) with 0.5 ml of $CaSO_4$ solution, as it is the way that gels are prepared. To prepare the relevant $CaSO_4$ solution concentration, we plotted the following abacus

$$n_{CaSO_4} = \frac{C_{p_{CaSO_4}} \cdot V}{M_{CaSO_4}}$$

where n_{CaSO_4} is the calcium quantity added into the gel, $C_{p_{CaSO_4}}$ the mass calcium concentration, V the solution volume used in the gel and M_{CaSO_4} the $CaSO_4$ molar mass.

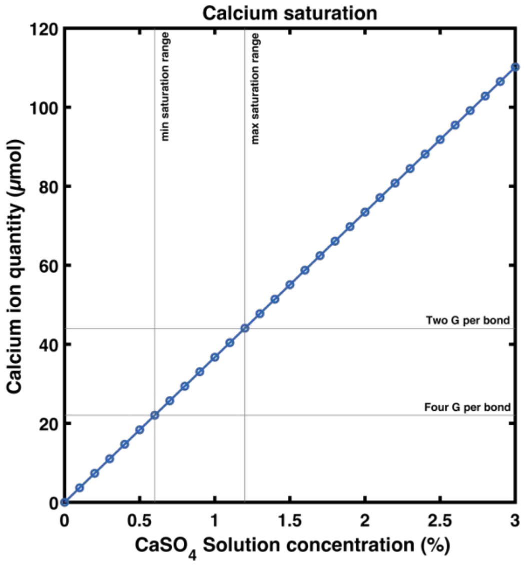

Figure S3:Calcium ion quantity present in a 2 ml of alginate 1.6 % (w/v) with 0.5 ml of $CaSO_4$ solution of different concentrations.

Compression experiments

Compression tests

Mechanical properties and stress stiffening phenomenon were quantified using an Optics 11 nano-indenter (Chiaro, Optics 11 For Life) and were run in a liquid environment (HBSS). The nano-indenter was mounted with a 100 µm radius spherical probe made of Polystyrene, E = 6 GPa and ν = 0.325 and with a cantilever rigidity of 0.5 N/m. Young modulus was obtained by fitting the force-displacement curve with a Ding linear elastic model (Equation S1), derived from the Hertz model (Equation S2) but applicable for larger deformations and hyperelastic materials:[5,6]

$$\mathrm{F} = \frac{4}{3}\mathrm{E}^{\star}\sqrt{\mathrm{R}}\xi^{3/2}\cdot\left(1-0.15\frac{\xi}{\mathrm{R}}\right)(S1)$$

$$F = \frac{4}{3}E^{\star\sqrt{R}}\xi^{3/2}(S2)$$

Where, F is the measured force (N), $E^{\star}$ the effective Young Modulus (Pa), R the radius of the indenting probe (m) and ξ the indentation (displacement, m).

However, the effective Young Modulus is defined as:

$$\frac{1}{E^\star} = \frac{1 - \nu_{Probe}^2}{E_{Probe}} + \frac{1 - \nu_{Sample}^2}{E_{Sample}}$$

Where ν_{Probe} and ν_{Sample} are the Poisson's ratios of the probe and of the sample respectively, and E_{Probe} and E_{Sample} are the Young's moduli of the probe and of the sample respectively. As E_{Probe} $\approx$ 3 GPa, and E_{Sample} is in the range of kPa, the effective young modulus can be re-written as

$$\frac{1}{E^\star} \approx \frac{1 - \nu_{Sample}^2}{E_{Sample}} \Rightarrow E^\star = \frac{E}{1 - \nu^2}$$

Where $E = E_{Sample}$ and $\nu = \nu_{Sample}$. As well as (S1 and S2) can be re-written as

$$F = \frac{4}{3}\frac{E}{1 - \nu^2}\sqrt{R}\xi^{3/2} \cdot \left(1 - 0.15\frac{\xi}{R}\right)(S3)$$

$$F = \frac{4}{3}\frac{E}{1 - \nu^2}\sqrt{R}\xi^{3/2}(S4)$$

We performed three complementary experiments on Alginate and Alginate + PEG gels. First, we set a baseline of the gel's mechanical properties by mapping the Young modulus every 5 µm on 100 µm distance. In addition, on the first indentation point (x=0), we also performed dynamic mechanical analysis to determine the rheological properties of the gel. Then, to simulate cell activity or repeated gel stimulation, we indented one hundred times on the first indentation point (x=0). Finally, to quantify the permanent modifications of the gel structure, we performed the same mapping as the first step. During indentations, location of the probe, displacement and force applied on the sample were recorded at a sampling rate of 1 kHz.

Young modulus calculations

Data was analyzed using Matlab (R2024a, MathWorks, USA). Contact point was automatically determined by the nano-indenter as a variation of 0.1 V signal variation corresponding to a displacement change about 5 nm from the baseline, and ν was considered equal to 0.5 due to the high presence of water composing the gel.[7] Then the ding model was fitted on all the force displacement signal using nonlinear regression model function of Matlab.

DSC experiments

To determine if Alginate and PEG interact with each other, we conducted Differential Scanning Calorimetry (DSC). If both materials interact with each other, their glass transition temperature T_g should be a combination of both alginate T_{g_A} and T_{g_P}.[8,9] To do so, we prepared Alginate 1.6 % (w/v) gels and Alginate 1.6 % (w/v) - PEG 0.32 % (w/v) gels both crosslinked with $CaSO_4$ (corresponding to 0.2 PEG to Alginate mass ratio). Gels were lyophilized and ground into powder for DSC measurements. We also checked the thermal behavior of pure CM-PEG-CH and neat alginate. DSC experiments were done using a Mettler Toledo DSC3+ system. Here, 12 mg samples were heated in a range of -50 °C to 250 °C, with a heating rate of 10 °C per minute under nitrogen.

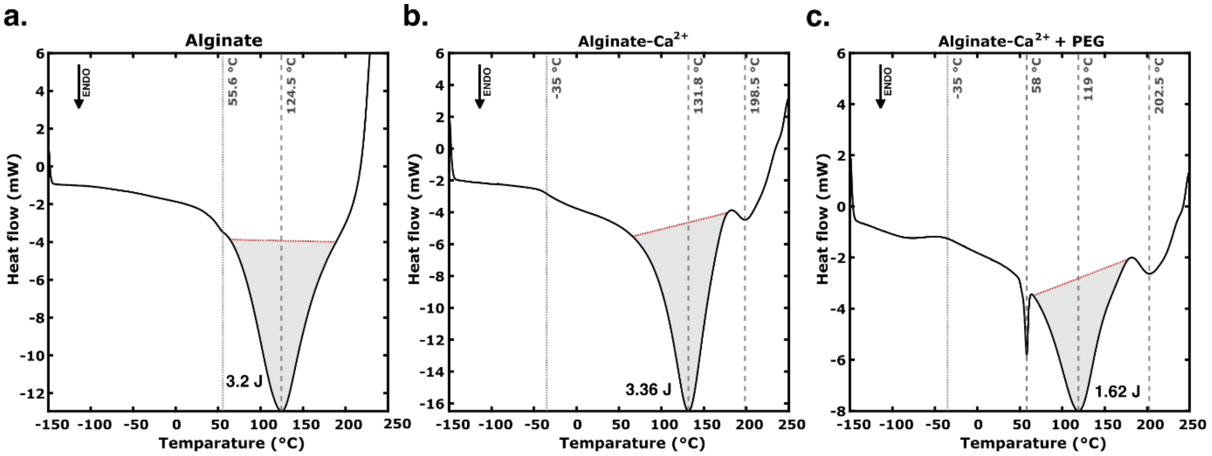

Figure S4: DSC thermograms of Na-Alginate Kimica I-1G (a), Alginate-Ca^{2+} freeze dried gel (b) and Alginate-Ca^{2+}+ PEG freeze dried gel (c). Dotted line corresponds to the glass transition temperature, dashed line corresponds to the melting temperature and greyed area corresponds to the endotherm integrals.

Melting points T_m and glass transition temperature T_g were determined respectively by finding with peaks on the first and second derivative of the heat-flow. Data were analyzed on Matlab 2024a. Regarding neat Alginate, T_{g_A} is 55.2 °C and there is a wide endothermic peak from 52 °C to 198 °C, centered at 112 °C. Previous reports found similar T_{g_A} and explain this endotherm as a mix between relaxation enthalpy and vaporization of water molecules attached to alginate chains.[8,10] Concerning freeze dried calcium alginate gel (**Figure S4b**), the Tg drops to -35 °C but the endotherm does not changes drastically, it is now centered to 131.8 °C and its integral remains in the same range as neat alginate (3.2 J instead of 3.36 J) finally, when PEG is added (**Figure S4c**), the endotherm slightly shifts to 119 °C but its integral is significantly minored to 1.62 J instead of 3.36 J. As the integral of the endotherm, can be understood the loss of bound water associated with the polymer change, it implies that PEG may reduce the water binding affinity to the alginate network which signifies easier release of bound water and thus a better water mobility in the network.[11]

Swelling experiment

The swelling ratio was calculated by placing cylinders (n = 4, 6 mm height, 9 mm diameter) of Alginate and Alginate-PEG gel into 2 ml of double distilled water during 24 h. Each cylinder was weighed before (t=0) and after 24 h of immersion (t=24h) on an analytical scale (# 321, Precisa, Switzerland), taking care of wiping the excess water with a Kim wipe. Cylinders diameter and height were also and measured, using a digital caliper with 2 digits accuracy. The swelling mass ratio Q_w was determined as the ratio of the weight after 24 h immersion ($m_{t=24}$) with the weight before immersion, $m_{t=0}$), and the volume swelling ratio Q_v was determined as the ratio of the volume after 24h immersion ($V_{t=24}$) with the volume before immersion ($V_{t=0}$). $V = \pi \cdot d/2 \cdot h$, where d is the cylinder diameter, and h the cylinder height.

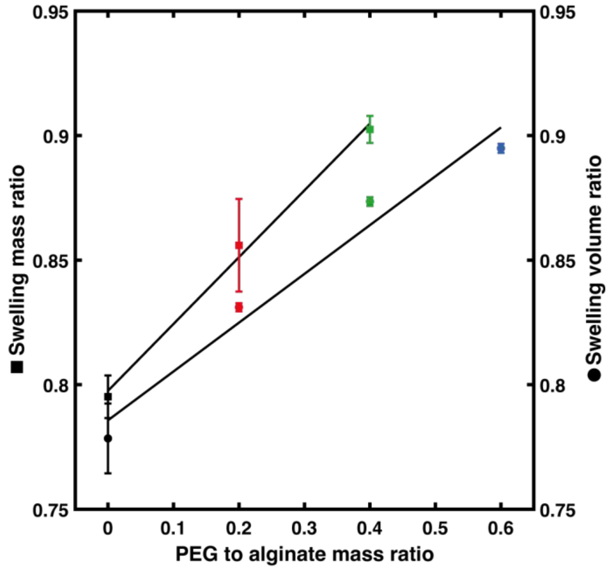

Figure S5: Equilibrium swelling mass and volume ratios for Alginate-PEG gel with PEG concentration varying from 0 to 0.8 PEG to Alginate mass ratio after 24 h immersion on water.

Alginate pore size determination

Wet gels - SAXS experiments

Wet samples were mounted on a vertical transmission SAXS mount and placed into a 9 kW XRD machine (Smartlab X-ray diffractometer, Rigaku Corporation, Japan) piloted with Smartlab Guidance version 2.0.2.5 (Rigaku Corporation, Japan). Measurements were performed in air, using a copper source (CuKα) with a tube voltage of 35 kV and a tube current of 150 mA.

Sample signal and background were recorded up to 2θ = 8 ° and analyzed between 0.2 and 2 °, the length of the background signal. After removing the background, pore size was determined using NANO-solver 3 fitting tool (version 3.7.6.0, Rigaku Corporation, Japan).

Pores were considered spheroid with a gamma distribution of diameter; matrix was set as alginate (d = 1.08 g/ml) and the measured geometry set as water (d = 1.00 g/ml) for pure alginate gel and as PEG (d= 1.13 g/ml) for alginate-PEG gels.

Confirmation of the method on dry gels

Freeze-drying

After molding, gels were placed at -20 °C overnight and froze-dried drying 12 hours using a Labconco Freezone 2.5 lyophilized, at a pressure of 0.014 mPa and at a temperature of -40 °C.

SAXS measurements

Dried films were placed in a Smartlab X-ray diffractometer, Rigaku, XRD machine piloted by SmartLab Guidance, Rigaku Corporation, Japan, Ver. 2.0.2.5.

To confirm our findings with SAXS, we wanted to perform direct measurement of the pores via electronic microscopy.

Electro-conductive coating

To increase gel electron conduction and thus have a better image resolution, gels were coated with evaporated carbon using a Q 10T ES plus, Quorum Turbomolecular pumped coater. Carbon was evaporated for 30 s at an evaporation current of 36 A.

Electron microscopy

Samples were observed using an Ultra-Plus FEG-SEM scanning electronic microscope in low vacuum mode (Zeiss, Germany).

Results

Both methods SAXS measurement on dried gels and SEM on dried gels led to the same average pore size about 17 nm, confirming the pore size determination method. We consider that this method is also applicable to wet samples.

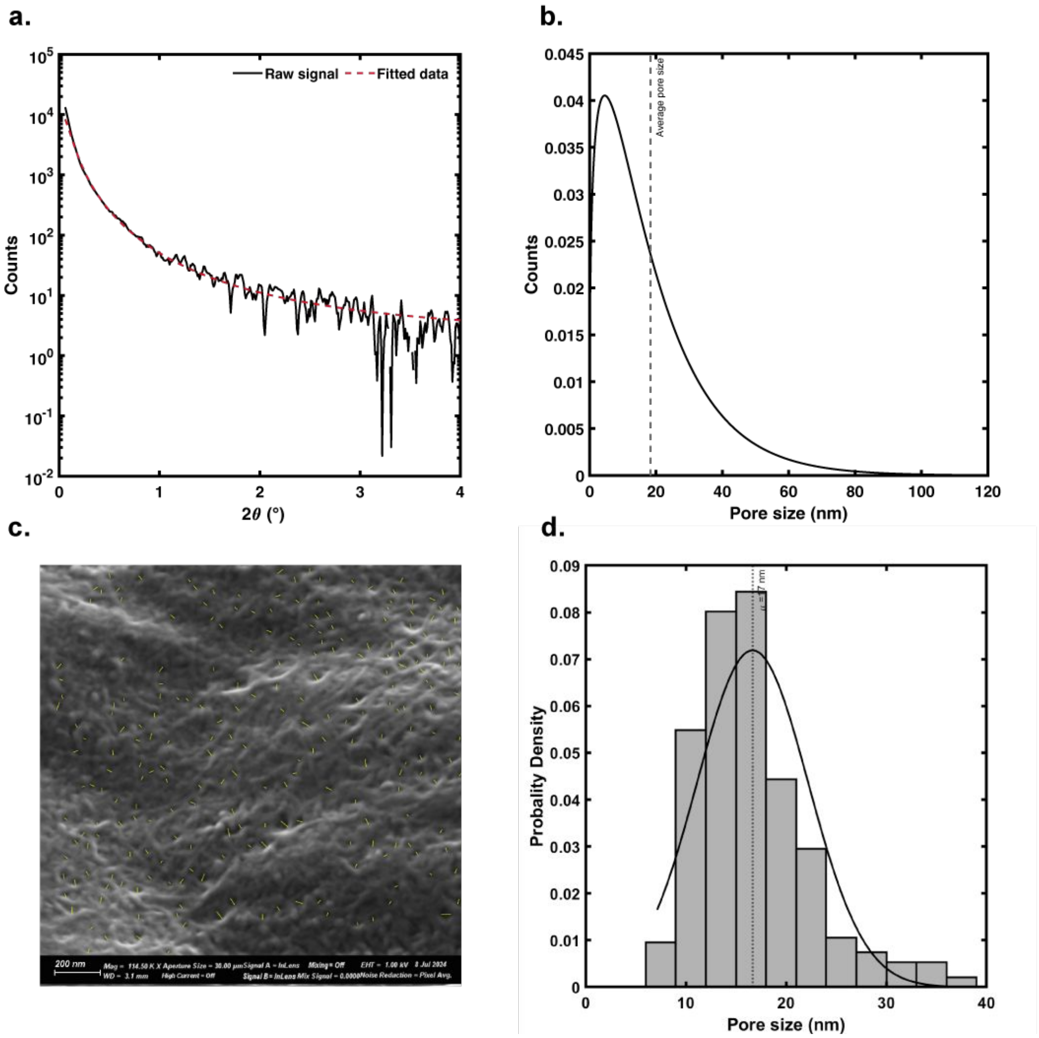

Figure S6: (a) raw SAXS spectrum used for pore size determination, (b) pore size distribution obtained after fitting the SAXS data, (c) SEM micrograph of freeze-dried Kimica I-1G 1.6 % (w/v) crosslinked with $CaSO_4$ 3 % (m/v), and (d) pore size distribution measured on the SEM.

Effect of PEG concentration on strain-stiffening phenomenon

Effect of indentation speed on mechanical properties

It is commonly accepted that the speed of indentation influences the determination of Effective Young's modulus over cyclic loading. Previous literature has shown that Effective Young modulus obtained by Hertzian fitting varies with the indentation rate to a certain extent following a root law.[12] They reported that after a critical speed, the Effective Young modulus became independent from the indentation speed, suggesting that the material was probed in its elastic domain. In addition, to avoid any relaxation phenomenon during the indentation cycle itself, the indentation speed should be at an indentation cycle shorter in duration than the relaxation time (about 5 s, **Figure S7**). To determine the adequate indentation speed, we should use for our experiment, we compared the average effective Young's Moduli obtained with different indentation speeds (1, 5 and 10 µm s^{-1}). We found that from 5 µm s^{-1}, the effective Young's Moduli were independent from the indentation speed, and thus decided to keep this indentation speed for all the tests.

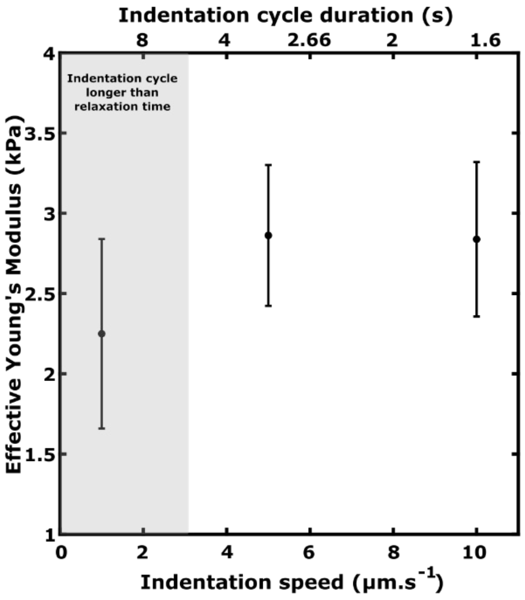

Figure S7 – Effective Young's Moduli as a function of the indentation speed

To see if PEG concentration influences the strain-stiffening stiffening phenomenon that occurred when we indent one hundred times at the same location the alginate gel, we tested different loading of PEG, from 0.2 to 0.6 PEG to alginate mass ratio. **Figure S8** clearly shows that the presence of PEG cancels the strain-stiffening phenomenon as after 100 indentations, the gel stiffened less than 2 % instead of nearly 10 % without PEG. Interestingly, the cancellation of the strain-stiffening phenomenon is independent from the PEG concentration, showing that a small quantity of PEG is enough to modify significantly the material mechanical properties.

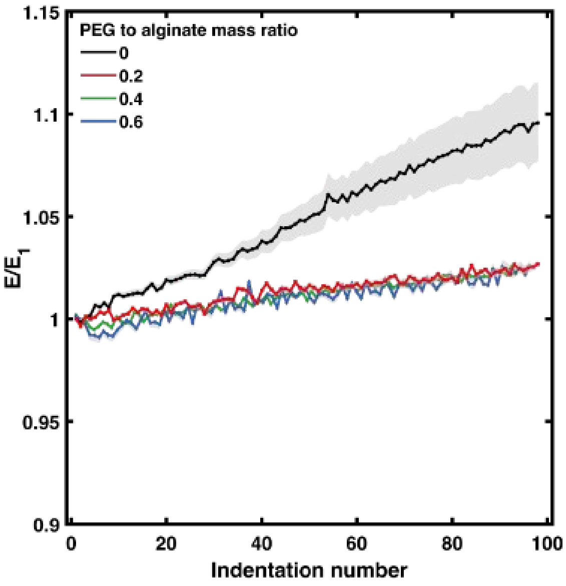

Figure S8: Stiffness ratio (E/E1) evolution over 100 immediate indentation cycles at a same location for pure alginate and alginate:PEG 0.2, 0.4 and 0.6 PEG to alginate mass ratio.

Relaxation tests

Relaxation tests were directly performed on gels after compression test. The test consists of maintaining the cantilever at the max displacement (10 µm) and measuring resultant force over-time as the gels relax. The measuring time was 30 s. Relaxation behavior of hydrogels can be described with a maxwell model:

$$F(t) - F_\infty = F_0 e^{-t/\tau}$$

where F(t), is the measured force at t time (N), F_∞ is the residual force at the `infinite', F_0 the measured force at the beginning of test (t=0), t the time (s), and τ is the relaxation time (s^{-1}). The model was fitted to the data using the nonlinear model fitting function of Matlab.

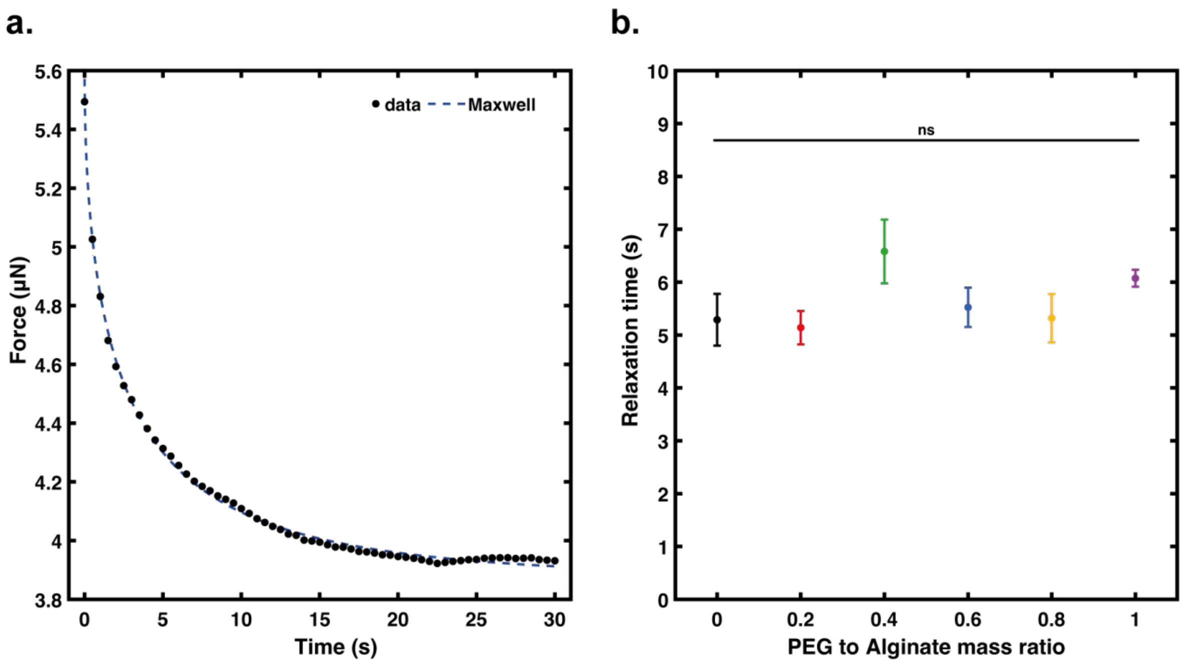

Figure S9: (a) Representative force relaxation test and its fitting with a one element Maxwell model. (b) Average Relaxation time over the PEG to Alginate mass ratio. for Alginate-PEG gel with PEG concentration varying from 0 to 0.8 PEG to Alginate mass ratio. No significant difference has been shown.

References

(1) Grolman, J. M.; Weinand, P.; Moone, D. J. Extracellular Matrix Plasticity as a Driver of Cell Spreading. *Proc Natl Acad Sci U S A* **2020**, 117 (42), 25999–26007. https://doi.org/10.1073/pnas.2008801117.

(2) Tortiglione, C.; Quarta, A.; Malvindi, M. A.; Tino, A.; Pellegrino, T. Fluorescent Nanocrystals Reveal Regulated Portals of Entry into and between the Cells of Hydra. *PLoS One* **2009**, *4* (11) e7698. https://doi.org/10.1371/journal.pone.0007698.

(3) Sartori, C.; Finch, D. S.; Ralph, B.; Gilding, K. Determination of the Cation Content of Alginate Thin Films by FTi.r. Spectroscopy. *Polymer* **1997**, *38* (1), 43–51. https://doi.org/10.1016/S0032-3861(96)00458-2.

(4) Silva, T. L. da; Vidart, J. M. M.; Silva, M. G. C. da; Gimenes, M. L.; Vieira, M. G. A. Alginate and Sericin: Environmental and Pharmaceutical Applications. In *Biological Activities and Application of Marine Polysaccharides*; InTech, **2017**. https://doi.org/10.5772/65257.

(5) Ding, Y.; Xu, G.-K.; Wang, G.-F. On the Determination of Elastic Moduli of Cells by AFM Based Indentation. *Sci Rep* **2017**, 7 (1), 45575. https://doi.org/10.1038/srep45575.

(6) Hertz, H. Ueber Die Berührung Fester Elastischer Körper. *J. fur Reine Angew. Math.* **1882**, (92), 156–171. https://doi.org/10.1515/crll.1882.92.156.

(7) Feliciano, A. J.; Grant, R.; Fernández-Pérez, J.; Giselbrecht, S.; Baker, M. B. Introducing Dynamicity: Engineering Stress Relaxation Into Hydrogels Via Thiol-Ene Modified Alginate for Mechanobiological in Vitro Modeling of the Cornea. *Macromol Biosci* **2024**, *24* (1) 2300109. https://doi.org/10.1002/mabi.202300109.

(8) Flores-Hernández, C. G.; Cornejo-Villegas, M. de los A.; Moreno-Martell, A.; Real, A. Del. Synthesis of a Biodegradable Polymer of Poly (Sodium Alginate/Ethyl Acrylate). *Polymers* **2021**, *13* (4), 1–12. https://doi.org/10.3390/polym13040504.

(9) Pereira Espíndola, S.; Norder, B.; Koper, G. J. M.; Picken, S. J. The Glass Transition Temperature of Heterogeneous Biopolymer Systems. *Biomacromolecules* **2023**, *24* (4), 1627–1637. https://doi.org/10.1021/acs.biomac.2c01356.

(10) Infanta Diana, M.; Selvasekarapandian, S.; Selvin, P. C.; Krishna, M. V. A Physicochemical Elucidation of Sodium Perchlorate Incorporated Alginate Biopolymer: Toward All-Solid-State Sodium-Ion Battery. *J Mater Sci* **2022**, 57 (17), 8211–8224. https://doi.org/10.1007/s10853-022-07185-w.

(11) Yang, X.; Dargaville, B. L.; Hutmacher, D. W. Elucidating the Molecular Mechanisms for the Interaction of Water with Polyethylene Glycol-Based Hydrogels: Influence of Ionic Strength and Gel Network Structure. *Polymers* **2021**, 13 (6). https://doi.org/10.3390/polym13060845 .

(12) Simič, R.; Mathis, C. H.; Spencer, N. D. A Two-Step Method for Rate-Dependent Nano-Indentation of Hydrogels. *Polymer* **2018**, 137, 276–282. https://doi.org/10.1016/j.polymer.2018.01.017.
